# Mapping healthcare leadership interventions and their performance in sub-Saharan Africa

**DOI:** 10.4102/jphia.v16i1.754

**Published:** 2025-03-10

**Authors:** Magome A. Masike, Ozayr Mahomed

**Affiliations:** 1 Department of Public Health Medicine, University of KwaZulu-Natal, Durban, South Africa; 2 Health Professional Council of South Africa, Pretoria, South Africa

**Keywords:** leadership, management, health, health system, outcomes

## Abstract

**Background:**

Healthcare leadership development programmes (LDPs) are gaining recognition globally as enablers for competent leadership. Sub-Saharan Africa countries are also implementing healthcare leadership development initiatives.

**Aim:**

This scoping review sought to map healthcare leadership interventions and their performance in sub-Saharan Africa.

**Setting:**

Sub-Saharan Africa.

**Method:**

A search of relevant articles was performed in PubMed, Semantic Scholar, and Academia, for articles written in English and published between 2003 and 2023. The Arksey and O’Malley framework was used to map the published studies. The Preferred Reporting Items for Systematic Reviews and Meta-Analyses extension for Scoping Reviews were used to report the results (PRISMA-ScR).

**Results:**

One hundred and ten articles were retrieved. Twenty-eight articles were included in the review. Twenty-one per cent (*n* = 6) of the studies on LDPs were conducted in South Africa. Twenty-one per cent (*n* = 6) used the ‘case study’ design, 18% mixed-method (*n* = 5) and 14% (*n* = 4) used qualitative study designs. Twenty-three of the 46 countries in sub-Saharan Africa conducted LDPs. Four programmes are postgraduate university degrees with entry requirements, while two programmes do not have any formal entry requirements

**Conclusion:**

Healthcare LDPs exist in sub-Saharan Africa. However, they are marketed as ‘healthcare leadership development’, while their content is management development.

**Contribution:**

This article summarises the research on the state and contributions of the healthcare LDPs in sub-Saharan Africa. Development of all future healthcare LDPs must consider applicable policies and be based on curricula that are focused on healthcare leadership competency development across all functional areas in the healthcare service delivery value chain.

## Introduction

Healthcare systems are widely acknowledged as ‘very complex’ to manage and lead.^1,2^ Healthcare leaders face multifaceted challenges, such as rising demands from informed patients, inadequate funding, inadequate workforce, and economic as well as ethical dilemmas.^3^ Adapting to an ever-changing healthcare environment, keeping up-to-date with the latest information, leading by example, being creative and innovative,^4^ and enabling a culture of communication, social exchange, creativity, and innovation are some of the necessary traits to lead the current healthcare environment into the future. Leaders in this environment will have strong communication skills and be scholars, health advocates, collaborators and professionals.^5^ The absence of positive leadership can result in a demotivated workforce, leading to poor health outcomes.^6^

The concepts of management and leadership are, at times, used interchangeably. Definitions of these concepts become critical to obviate misunderstandings when these are encountered in this text. Management involves planning, leading, organising and controlling organisational resources (human, financial, material). Leadership is a process whereby individuals can influence others to achieve or reach commonly desired outcomes.

Healthcare leadership development is complex and has received attention since the 1960s, given impetus by the Griffiths report in 1983.^7^ Thus, it has received significant and constant attention globally since then. The World Health Organization (WHO) is at the forefront of these developments and has encouraged countries, continents and economic blocs to customise healthcare leadership development to be context-informed.^8^ The healthcare sector started developing and implementing leadership development models and frameworks to influence and improve health system outcomes.

The often-cited healthcare leadership models and frameworks adopted and currently in implementation in different geographies across the world include the National Health Service’s (NHS) Leadership Model in the United Kingdom,^9^ the European Foundation for Quality Management (EFQM),^10^ the Canadian Health’s Lead Self, Engage Others, Achieve Results, Develop Coalitions and Systems Transformation (LEADS) Capability Framework,^11^ the Australia Health Leadership Framework,^12^ and others.

Healthcare leadership development in sub-Saharan Africa is also gaining attention, and conversations have commenced through conferences and country-specific research on this as one of the instruments that can be used to improve health system performance and outcomes.^13^

The challenges of the healthcare systems of countries in sub-Saharan Africa and/or Africa can be traced to the need for more, better or varied levels of healthcare leadership development initiatives in implementation on the continent. This has contributed to poor health outcomes in sub-Saharan Africa and has highlighted the need for healthcare leadership development interventions to improve health system performance and outcomes.

In contrast to the previous scoping review assessing interventions on what leadership capabilities are most important or how effective different leadership development models are,^14^ this study mapped out the available healthcare leadership interventions to determine the country and regions where they are in implementation on the continent to determine coverage, the curricula, the educational levels of the leadership development programmes (LDPs) in terms of the national qualification’s framework. ‘Good practices’ such as EFQM, NHS, Canadian LEADS and the Australian LEADS are used as a frame of reference when assessing the depth and breadth of the LDPs.

This scoping review sought to map healthcare leadership interventions implemented in sub-Saharan Africa, determine their performance where applicable and assess whether monitoring and evaluation, and performance management are included in the current LDPs.

## Methods

This scoping literature review was performed according to the Arksey and O’Malley methodological framework^15^ to map the evidence from academic literature published over the last 20 years (2003–2023) on healthcare leadership development in sub-Saharan Africa. The Preferred Reporting Items for Systematic Reviews and Meta-Analyses extension for Scoping Reviews (PRISMA-ScR) were used to report the results.^16^

The following questions formed the basis for the literature search:

- *Which healthcare LDPs were and are in implementation in sub-Saharan Africa?*

The following lower-level questions flow from the first research question:

- *What is in the curriculum of the identified healthcare LDPs?*
- *Who is the target population of the healthcare LDPs?*
- *What is the impact of the identified healthcare leadership development on the health systems of countries in sub-Saharan Africa?*
- *What must be done to get the healthcare leadership development initiatives to achieve outcomes comparable to what other geographies are experiencing?*

A search for relevant articles was performed in PubMed, Semantic Scholar, Academia and other databases. Articles, journals and books that addressed the research questions were identified using the following search terms: ‘Healthcare leadership development in sub-Saharan Africa’, ‘Healthcare care leadership development in sub-Saharan Africa’, ‘Healthcare leadership development in Africa’, ‘Health care leadership development in sub-Saharan Africa’, ‘Healthcare leadership development initiative in sub-Saharan Africa’, ‘health care leadership development initiative in Africa’, ‘healthcare leadership development initiative in Africa’, ‘health care leadership development initiative in Africa’, ‘health care leadership development programme in Africa’, ‘healthcare leadership development programme in Africa’, ‘healthcare leadership development programme in sub-Saharan Africa’ or ‘health care leadership development programme in sub-Saharan Africa’. This literature search was restricted to articles written in English and published between 2003 and 2023. Articles with a general leadership focus and from a public administration perspective were excluded. The scoping review incorporated qualitative research systematics and meta-analyses.

### Search strategy

Joanna Briggs Institute members and five Joanna Briggs Collaborating Centres recommended using the population, concept and context (PCC) approach to narrow the review’s focus and scope^17^ (Table 1).

**TABLE 1:** Population, concept and context framework.

PCC Element/Criteria	Criteria/determinant		
P – Population	-		
C – Concept	‘healthcare leadership development in sub-Saharan Africa’ OR ‘health care leadership development in sub-Saharan Africa’ OR ‘healthcare leadership development in Africa’ OR ‘health care leadership development in sub-Saharan Africa’ OR ‘Healthcare leadership development initiative in sub-Saharan Africa’ OR ‘health care leadership development initiative in Africa’ OR ‘healthcare leadership development initiative in Africa’ OR ‘health care leadership development initiative in Africa’ OR ‘health care leadership development programme in Africa’ OR ‘healthcare leadership development programme in Africa’ OR ‘healthcare leadership development programme in sub-Saharan Africa’ OR ‘health care leadership development programme in sub-Saharan Africa’.		
C – Context	Angola	Ethiopia	Niger
	Burkina Faso	Gabon	Nigeria
	Burundi	Gambia, The	Rwanda
	Cabo Verde	Ghana	Sao Tome and Principe
	Cameroon	Guinea	Senegal
	Central African Republic	Guinea-Bissau	Seychelles
	Benin	Kenya	Sierra Leone
	Botswana	Lesotho	Somalia
	Chad	Liberia	South Africa
	Comoros	Madagascar	South Sudan
	Congo, Democratic Republic of	Malawi	Sudan
	Congo, Republic of	Mali	Tanzania
	Cote d’Ivoire	Mauritania	Togo
	Equatorial Guinea	Mauritius	Uganda
	Eritrea	Mozambique	Zambia
	Eswatini (Formerly Known as Swaziland)	Namibia	Zimbabwe

#### Selection process, data extraction and synthesis

The principal investigator retrieved all relevant literature from the databases and duplicate titles were removed. Two researchers independently reviewed each title and abstract to identify relevance to the aim and question of the current study. The researchers reviewed the titles, abstracts and full content of the articles that were omitted or included in terms of relevance to the study question. The researchers agreed on which articles should be included in a full review. A predefined Microsoft (MS) Excel sheet charting form was used to extract the data from the complete articles of the selected articles. The predefined columns included author, year of publication, aim of the study, study design, primary findings, and study setting. A documents folder was created on the researcher’s hard drive to store all articles found to be relevant to the research. The articles identified as entirely relevant were also loaded to Mendeley for automatic citation and reference list generation. Data were extracted into an MS Excel table.

Emerging synthesis was used in this qualitative research, which included both theoretical works and relevant grey literature. Tables, graphical displays and charts were used to enable the synthesis to be possible.

The charted data were split into key thematic sections including programme structure, learning content and learning methods. Data were grouped into common categories and summarised in the tables. The findings were then analysed and synthesised in the context of the overall review questions and specified study objectives.

#### Consultation with stakeholders

No external stakeholders were involved in identifying the topic or deciding regarding the articles reviewed. However, the results will be presented to an external forum or policymakers.

## Results

**FIGURE 1:**
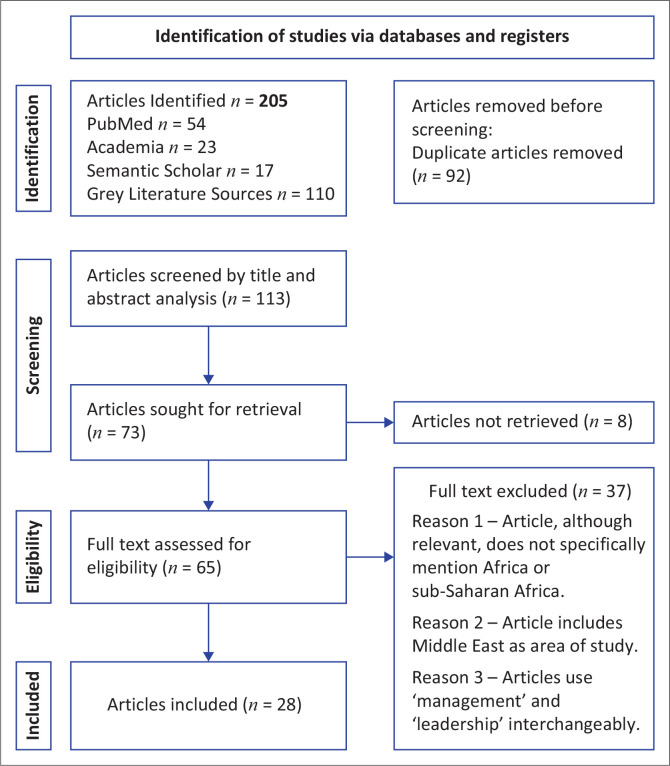
Preferred Reporting Items for Systematic Reviews and Meta-Analyses (PRISMA) flow diagram showing literature search and selection process.

The data collation from the three mentioned databases yielded 205 articles. After the duplicate records were removed, 112 records were screened in line with the main research question. Of these, 65 studies fulfilled the inclusion criteria and were included in the review stage. Twenty-eight articles were identified as relevant. A summary of the study selection process is shown in Figure 1.

Of the 28^13,14,18,19,20,21,22,23,24,25,26,27,28,29,30,31,32,33,34,35,36,37,38,39,40,41,42,43,44^ articles included in the review, the distribution by year of publication is as follows: 2012 (4%, *n* = 1),^13^ 2014 (7%, *n* = 2),^18,29^ 2015 (4%, *n* = 1),^39^ 2016 (4%, *n* = 1),^4^ 2017 (7%, *n* = 2),^31,40^ 2018 (7%, *n* = 2),^30,38^ 2019 (7%, *n* = 2),^31,37^ 2020 (4%, *n* = 1),^14^ 2021 (18%, *n* = 5),^6,23,25,36,44^ 2022 (11%, *n* = 3),^26,33,35^ 2023 (21%, *n* = 6)^11,22,32,41,43,45^ and 2024 (4%, *n* = 1).^42^ Twenty-one per cent (*n* = 6) of the studies on LDPs were conducted in South Africa followed by Uganda (11%, *n* = 3), Kenya (11%, *n* = 3) and Zambia (11%, *n* = 3). Eight other countries contributed to the remaining 25% (*n* = 7) of articles (Table 2).

**TABLE 2:** Descriptive characteristics of the final included studies.

Study number	Author	Year of publication	Aim of study	Findings of study	Study design	Setting
1.	Doherty et al.^17^	2014	To evaluate the Oliver Tambo Fellowship Programme as a health leadership training programme.	The Diploma offered a unique contribution in that it sought to empower and galvanise students to become change agents in the complex settings of their workplaces.	A rapid, descriptive study making use of mixed-method techniques. Document review. A brief questionnaire as well as semi-structured telephonic interviews.	South Africa
2.	Johnson et al.^13^	2020	To determine what the required leadership capabilities are most important or how effective different leadership development models are.	LDPs were undertaken in 23 of the 46 countries in sub-Saharan Africa. It was also established that one or two studies were carried out in each of the remaining eight countries.	Scoping review	Sub-Saharan Africa
3.	Chigudu et al.^18^	2018	The exploration of the concept and practice of healthcare leadership at sub-national level in a low-income country setting, using a people-centric research methodology.	Many of the participants stated, implicitly and explicitly, that the different leadership styles that they employ overlap continually and must be leveraged flexibly depending on the demands of the moment or the task at hand.	In-depth interviews	Gambia
4.	Agyepong et al.^19^	2017	This article presents findings of the research to verify relevance, identify competencies and support programme design and customisation.	There were several non-degree short courses and a few degree programmes with some leadership capacity building focus. For example, (1) The Ghana Institute of Management and Public Administration (GIMPA) – executive Masters in Governance and Leadership and PhD in Governance, Leadership and Public Administration (2) Almond Institute, Kwame Nkrumah University of Science and Technology (KNUST) – The Graduate School of Governance and Leadership (GSGL) – BSc International Business Administration and Global Leadership. (3) South Africa (Business and Public Management Schools) – Postgraduate diploma and Master’s level programmes. (4) 4A University of Cape Town – Postgraduate Diploma in Health Management (Oliver Tambo Fellowship Programme). 4B Albertina Sisulu Executive Leadership Programme in Health (ASELPH) offered at postgraduate Diploma and MPH levels. (5) Makerere University – 2-year non-degree fellowship programme for those working in government or the private sector. (6) Makerere University Business school – Master of Science in Leadership and Governance. (7) Uganda Christian University – Master’s degree in Public Health Leadership, targeted at improving maternal health.	The study was a multiple case study of Ghana, South Africa, and Uganda. In each case, the unit of analysis was the same. Cross-sectional mixed-methods of data collection were undertaken in all three countries.	Sub-Saharan Africa
5.	Mathole et al.^20^	2017	This article reports on a subsequent case study undertaken to examine leadership practices and the functioning of maternal health services in two resource-limited hospitals with disparate maternal health outcomes.	Four key themes emerged from the analysis. The first theme relates to external context and infrastructure, including the history and formal structure of the two hospitals in the district health system. In this theme, findings related to leadership and management of equipment, supplies and infrastructure were closely linked to the material and structural conditions in the hospitals and district rather than leadership itself being identified as the primary issue or driver.	Mixed-methods – case study design was used. Semi-structured interviews.	South Africa
6.	Ikhile et al.^21^	2023	This study thus explores need-based leadership training needs for pharmacists to provide effective AMS and inform the CPA’s development of a focused leadership training programme.	Data analysis revealed a clear need for a health leadership programme, with 61% of respondents finding previous leadership training programmes highly beneficial or just beneficial.	A mixed-methods approach. Quantitative data were collected via a survey across eight sub-Saharan African countries. Qualitative data were collected.	Sub-Saharan Africa
7.	Tukuru et al.^22^	2021	The aim of this study was to assess the need for leadership and management training in Dentistry in South Africa from an industry perspective.	Participants stated that strong leadership and managerial skills were important for dealing with these challenges.	A qualitative study – purposive sampling. One-on-one and telephonic interviews guided by a semi-structured questionnaire with open-ended questions.	South Africa
8.	Chelagat et al.^23^	2021	To explore the impact of leadership training on health system performance in selected counties in Kenya.	Trained healthcare management teams had a significant difference in the implementation status of priority projects and hence had a significant impact on health system performance indicators compared to non-trained managers.	A quasi-experimental time series design. Pretest and post-test control group design was utilised. Questionnaires were administered to trained health managers.	Kenya
9.	Nzinga et al.^24^	2021	To determine content and co-development of a participatory intervention at building leadership skills.	Following training, managers reported greater recognition of the importance of health system values, belief systems and relationships and improved self-awareness.	Reflective engagements between researcher and decision-makers	Kenya
10.	Dimbuene et al.^25^	2022	To understand the role of leadership at regional, national and local levels to improve mother and child health outcomes in sub-Saharan African countries.	There is an urgent need to focus on research and development and innovation. To achieve this goal, a crucial shift in leadership is compulsory.	None articulated	Sub-Saharan Africa
11.	Curry et al.^12^	2012	To characterise the experiences of individuals in key healthcare leadership roles in sub-Saharan Africa.	Five themes emerged:- Aspirational value based vision; Being self-aware Ability to identify and use complementary skills of others Tending to relationships Using data in decision making	Qualitative study Interviews held Data analysis	Sub-Saharan Africa
12.	Azeez T.A^26^	2023	Examine how each leadership theory could be applied to advance healthcare delivery in sub-Saharan Africa.	Different leadership styles have associated merits and demerits, but the hybrid approach that analyses relevant variables would be the optimal approach to reinvigorate healthcare delivery	Not articulated	Sub-Saharan Africa
13.	Monroe-Wise et al.^27^	2016	To assess what career changes if any the Afya Bora Fellowship’s alumni have experienced since completing the fellowship and to describe those changes.	All alumni reported improved performance at work and cited the application of a wide range of fellowship skills including leadership, research, communication and mentoring	Electronic survey administered	Botswana; Kenya; Tanzania and Uganda
14.	Kwamie^28^	2014	To address how and why the leadership development programme (LDP) ‘works’ when it is introduced into a district health system in Ghana, and whether or not it supports systems thinking in district teams.	The LDP was a valuable experience for district managers and teams were able to attain short-term outcomes because the novel approach supported teamwork, initiative-building and improved prioritisation. However the LDP was not institutionalised in district teams and did not lead to systems thinking.	Realist evaluation; Explanatory case study; Participant observation; Document review and semi-structured interviews	Ghana
15.	Govender et al.^29^	2018	Gain comprehensive understanding of the challenges, complexities and constraints facing public healthcare in KwaZulu-Natal (KZN) and to examine leadership as a strategy to enhance healthcare service delivery with a focus on four regional hospitals in the KZN province.	The current leadership framework adopted by the healthcare leaders in regional hospitals in KZN is weak and is contributing to poor healthcare service delivery.	Mixed-method research	KwaZulu-Natal province, South Africa
16.	Sammut and Ngoye^30^	2019	The development, structure and innovation of the Healthcare Management MBA programme	Students to the programme have requested more content on leadership, integrating traditional medical care and belief systems into allopathic care, supply chain management and in-depth review of the producer function and its relationship with providers	Not articulated	Kenya
17.	Mubuuke et al.^31^	2023	To describe the programme’s implementation process, share the experiences of participants and discuss lessons learned	Participants reported increased knowledge, skills, and confidence in attaining key leadership competencies.	Surveys	Uganda
18.	Wessels^32^	2021	To understand leadership development of appointed and emergent leaders in new medical schools in Africa.	A need for a development of a framework to navigate the complex nature of leadership development in new medical schools in Africa.	Mixed-methods approach; Multiple case study and qualitative document analysis	University of Cape Town, South Africa
19.	Malakoane et al.^33^	2023	Describes the process to implement and measure the effects of the Health systems Governance and Accountability (HSGA) intervention for system-wide improvement of leadership/management under routine conditions in a resource-constrained setting.	The mean scores on three Balanced Scorecard perspective improved statistically from year on year.	Normalisation process theory; Participatory discussion	Free State province, South Africa.
20.	Teame et al.^34^	2022	To assess the effectiveness of healthcare leadership and associated factors among managers working at public health institutions.	The challenges in healthcare leadership were mainly associated with a lack of leadership knowledge and skills.	Cross-sectional study triangulated with qualitative study. Random sampling method	Addis Ababa, Central Ethiopia
21.	Talbert-Slagle et al.^35^	2021	To describe the health management programme in Liberia, its focus and evolution from programme launch in 2017, to the present as well as ongoing efforts to transition programme activities to local partner ownership by the end of 2021.	The health management programme in Liberia can serve as a model for other efforts that seek to build health management capacity in resource-limited settings and enable local higher education institutions to offer locally appropriate, tailored educational programmes to strengthen health management system-wide	Case study	Liberia
22.	Pfeiffer et al.^36^	2019	The World Health Organization has identified leadership development as integral to achieving successful health outcomes, but few programmes exist for frontline healthcare workers in low-resources settings.	Thirteen participants developed a total of 17 leadership projects to apply their training.	A multi-modal training approach. An 18-month pilot leadership development programme	Ghana
23.	Forster et al.^37^	2018	To share the implementation outcomes of a Certificate in Leadership and Management Practice (CLMP) programme in rural settings	The facility heads had strengthened their leadership and management competencies.	Case study	Zambia
24	Nakanjako et al.^38^	2015	Share lessons from the Afya Bora Global Health Leadership Fellowship programme as implemented in Uganda	With structures mentorship, collaborative activities at academic institutions and local healthcare programmes equipped healthcare providers with leadership skills.	Case study	Uganda
25.	Mutale et al.^39^	2017	Presents the results of an impact evaluation of the Zambian Management and Leadership Academy programme conducted in 2014–2015	Leadership and management training will be a key ingredient in health system strengthening in low-income settings	Cross-sectional mixed-method study	Zambia
26.	Aminu et al.^40^	2023	To share the outcomes and impact of Leadership Development Academy in Nigeria	Capacity building can lead to skill development if well aligned with clearly defined organisational functions and based on clear theories of change and action that must be developed together with the critical stakeholders within the organisation.	Case study	Nigeria
27.	Mucheru et al.^41^	2024	The study is aimed at addressing the organisational and individual barriers to the advancement of women to leadership positions in the Tanzanian health sector and to evaluate the influence on leadership competencies and career advancement actions of the female health workforce.	The notion that progression towards gender equality in healthcare leadership is attained by fashioning a system that support the advancement of women.	Gender transformative approach	Tanzania
28.	Sekeseke^42^	2023	To understand the current practices of healthcare leadership development and determine authentic leadership programme that builds capacity and capabilities; the human related factors influence on leadership development and organisational factors that support leadership development.	That leadership development programme builds capacity and competencies in leaders, human factors are an important factor in leadership development and that the organisational structure and systems are critical factors in supporting leadership development; however, there is also the acknowledgement that the leadership development is not supported and leaders in healthcare organisations are not trained.	Multi-paradigm approach	Zambia
29	I Lutwama et al.^43^	2022	To explore the characteristics, barriers, and facilitators to the implementation of the Boma Health Initiative’s (BHI) Communities Health Workers (CHE) intervention in South Sudan between 2011 and 2019.	The coordination mechanisms among stakeholders have been weak, leading to wastage and duplication of resources. Duration of training of community health workers is short, and refresher trainings were rare.	Qualitative approach	South Sudan

Note: Please see the full reference list of the article Masike MA, Mahomed O. Mapping healthcare leadership interventions and their performance in sub-Saharan Africa. J Public Health Africa. 2025;16(1), a754. https://doi.org/10.4102/jphia.v16i1.754, for more information

PhD, Doctor of Philosophy; BSc, Bachelor of Science; MPH, Master of Public Health; MBA, Master of Business Administration; AMS, antimicrobial stewardship; CPA’s, Community Pharmacy Association.

Twenty-one per cent (*n* = 6) used the ‘case study’ design. Eighteen per cent (*n* = 5) utilised the mixed method, and 14% (*n* = 4) used qualitative study. A different author published each of the 28 articles. The articles were published between 2012 and 2024 (Table 2).

Leadership development programmes were undertaken in 23 of the 46 countries in sub-Saharan Africa. Most of these LDPs are unique to each country. Table 2 provides a summary of the various LDPs in sub-Saharan Africa. It was observed that most LDPs use ‘leadership’ and ‘management’ interchangeably.

### Healthcare leadership programmes and curriculum – Verification

Information on details of the type of LDPs was available for seven programmes. Four programmes are postgraduate university degrees with entry requirements, such as a Bachelor’s or Master’s degree in public health or, for the top-end qualification, a doctorate in public health. Two programmes do not have any formal entry requirements (Table 3).

**TABLE 3:** Summary of the entry requirements, location and aim of the leadership development programmes.

Number	Programme name	Setting/location	Level of programme	Minimum requirements	Target audience of programme	Summary of modules of the programme
1	Albertina Sisulu Executive Leadership Programme in Health (ASELPH)	South Africa	Executive level certificate	Three-year University Bachelor’s degree (NQF 7) and/or above. 4 Years working experience in Health sector.	Executive, district and hospital managers	The programme includes 10 modules covering management areas like strategic planning and leadership disciplines such as transformation and ethics
2	Doctor of Public Health (DRPH)	South Africa	Postgraduate level degree	Master’s degree in public health.	Public health professionals	The programme includes nine modules focusing on planning, systems theory, policy development, and leadership.
3	Doctor of Public Health (DRPH)	Uganda	Postgraduate level degree	Master’s degree in a relevant field	Any student meeting the entry requirements	The programme consists of nine modules which include building capabilities in planning, systems theory, policy development as well as leadership.
4	Enhancing Leadership, Management and Governance Competencies	Ethiopia	Postgraduate certificate level	Not applicable	Policy-makers and strategic decision makers; district, health centre and hospital managers and primary healthcare unit managers	The programme consists of four modules that focus on leadership and management development.
5	Afya Bora Consortium Fellowship	Kenya	Executive level certificate level	Citizenship in Kenya Medical applicants must have MD or MBCHB with Master’s degree in related field. Nursing applicants need to have a Master’s degree in nursing, Public Health or related field. Other public health professionals to have doctoral degree in Public Health or a related field	Any holders of MBCHB with Master’s degree. Nursing applicants with Master’s degrees in nursing or public health. Other public health professionals with doctoral degrees	The programme consists of nine modules that span the management disciplines such as monitoring and evaluation as well as leadership as a free standing course offering.
6	Zambia Management and Leadership Course for District Health Managers in Zambia	Zambia	Customised postgraduate certificate level	There are no formal prerequisites.	District health care managers	This programme consists of six modules that only address management competency gaps in areas such as project management.
7	Afya Bora Consortium Fellowship	Botswana	Executive level certificate level	Citizenship in Botswana Medical applicants must have an MD or MBCHB with Master’s degree in related field. Nursing applicants must have a Master’s degree in Nursing, public health or related field. Other public health professionals must have Doctoral Degree in Public Health or a related field.	Any holders of MBCHB with Master’s degree Nursing applicants with Masters degrees in Nursing or public health Other public health professionals with doctoral degrees	The programme consists of nine modules that span the management disciplines such as monitoring and evaluation as well as leadership as a free standing course offering. This programme does not include leadership as a taught module.
8	Doctor of Public Health (DRPH)	Ghana	Postgraduate level degree	Master’s degree (MPH/MPhil or its equivalent) in a relevant public health area.	Public health practitioners who are holders of public health Master’s degrees.	This programme consists of nine modules that are similar in nature with the Doctorate level programme offerings presented in South Africa and Uganda.

NQF, National Qualifications Framework; MPil, Master of Philosophy; MPH, Master of Public Health; MD, Doctor of Medicine; MBCHB, Bachelor of Medicine, Bachelor of Surgery.

The Albertina Sisulu Executive Leadership Programme in Health (ASELPH) from South Africa offers a 10-module curriculum^46^; the Enhancing Leadership, Management, and Governance Competencies programme from Ethiopia offers a 4-module curriculum^47^; the Zambia Management and Leadership Course for District Health Managers in Zambia is a programme offering 6-module curriculum^40^; and five programme offerings from South Africa, Botswana, Kenya, Uganda and Ghana have 9-module curriculum offerings.^14^

The Zambia Management and Leadership Course for District Health Managers^40^ is a programme that does not offer ‘leadership’ as a module. However, it is still a healthcare leadership development initiative. The modules in these programmes cover the following subject areas: Leadership, Strategic Management, Project Management, Financial Management, Communications, Planning, Health Systems, Transformation and others.

The modules on offer are usually offered in general management training. The healthcare LDPs discussed here were designed to attract those already in management. This identified target student population casts the ‘net narrowly’ while the intent is to develop healthcare leadership. This approach differs from what is done in other geographies.

## The impact of the implemented healthcare leadership development programmes

A total of 1414 students attended the seven healthcare LDPs reviewed. Overall, the participants were optimistic about the LDPs’ impact on their professional roles within the healthcare system.

The participants have seen increased knowledge, skills and confidence in key leadership competencies. In addition, the LDPs provided a systems view, which assisted in understanding the role and impact of the external context and the healthcare infrastructure. Some of the LDPs resulted in improvements in self-awareness for the attendees.^45^

The participants indicated that the programme structure needed improvement in terms of incorporating health system values, belief systems, and relationships, improved self-awareness and relationship management. Structured mentorship is integral to some LDPs, and the collaborative activities that must be implemented equip participants with leadership skills. Furthermore, the non-institutionalisation of LDP content hindered system thinking.^29^ From a health system perspective, participants indicated a need for more curriculum content on healthcare leadership, which must be inclusive or integrate traditional medical care. The LDPs must be designed to support or be part of a system that advances the aspirations of women working in the healthcare sector.^42^

## Discussion

This scoping review demonstrated that sub-Saharan African countries implement ‘Healthcare leadership development training initiatives and programmes’.

Fifty per cent (50%) of the reviewed articles (*n* = 14 out of 28) affirm a positive correlation between specific leadership training and improved leader performance outcomes in targeted healthcare systems. Additionally, two key observations were made: (1) healthcare leadership development initiatives lack standardisation, and (2) interventions in healthcare leadership encompass diverse formats, including non-degree certificates, degree programmes, postgraduate diplomas, Master’s degrees and doctoral courses, indicating a lack of uniformity. Some studies also explore suitable leadership styles for healthcare settings.

Further analysis showed that seven of the studies point to a need for healthcare LDPs that can emphasise areas such as: (1) leadership development for women in the healthcare system, (2) leadership development for those working in healthcare support services (e.g., infrastructure, supply chain management, healthcare technology, and others), and (3) making the healthcare leadership development initiative contextually relevant, with a focus on healthcare leadership development as the core training intervention.

The findings are that six different types and levels of healthcare LDPs are presented from West Africa, East Africa and Southern Africa. These offerings differ from country to country. The wide spread of the offerings points to complete coverage of healthcare leadership development in sub-Saharan Africa.

However, the variety of the programmes will, in the future, create sustainability challenges. The sustainability of health and social care systems and the support of professional staff to enable them to practise is an imminent future healthcare challenge.^45^

There are ‘healthcare leadership development’ programmes in sub-Saharan Africa, which are a platform for growth. However, these programmes are of different types and levels of healthcare leadership development, and no two or more countries are implementing the same programmes.

This review noted that the need, role, healthcare development approaches, healthcare leadership programmes, and curriculum verification should be collaborative. For example, in North America (United States and Canada), collaboration is being developed based on a matrix leadership structure.^4^ Globally, integrated care reports are needed, and policymakers highlight the importance of collaboration in organisations and among employees; continents and countries have different cultural values, spending priorities and funding streams within healthcare structures.

This review showed that the assessment of design, content, theory, practices and impact on healthcare service delivery positively affected the skills of the target student population. However, low-income countries have financial constraints that compromise the need for further healthcare leadership training and the inclusion of more modules to reach the standard of the developed world.^5^ Furthermore, the study also found that women’s leadership advancement aspirations and the importance of the totality of all healthcare system components must be supported.

Even though the programmes are marketed as ‘healthcare leadership development’, closer scrutiny of the curriculum shows that their focus is on ‘health care management development’ programmes instead. Leadership and management cannot be viewed as synonyms. In addition, initiatives to develop competencies in either leadership or management are different, and there are no overlaps. Furthermore, the study found improved leadership self-awareness and potential for further self-improvement.

There is a need to streamline the current ‘healthcare leadership development’ offerings and focus them on ‘leadership’ rather than ‘management’. Leadership is about motivating and inspiring organisations, whereas management is about using organisational resources to attain efficiency.

The studies indicate that practitioners and persons employed by the healthcare sector are eager to attend all the offered ‘leadership’ training, and there is overwhelmingly positive feedback on its impact. Redirecting the leadership training will create a network of people who will motivate and inspire the healthcare delivery system for better outcomes.

This study finds that sharing experiences and the impact of the implemented healthcare LDPs improves leaders’ knowledge, skills, confidence and competence. Effective healthcare leadership depends on training in the right skills and values and the development of agents of change.^48^ The rapid expansion of technology, a constantly changing disease profile, and accessibility to evidence, contrasted by the global health inequities, demonstrates the complexity of the required leadership and confirms the relevance of sharing information.

We must determine the contexts and needs for healthcare leadership development across sub-Saharan Africa. Leadership development should not be restricted to the few in or close to the Executive Management ranks.

Healthcare LDPs are generic because the curricula focus on subjects easily provided in other programmes, such as strategic planning, communication and human resources management. This confirms the argument that with many LDPs, the assumption is that ‘one size fits all’. Furthermore, when attention is not sufficiently paid to the participant’s baseline inventory of skills and developmental goals, the focus on the behaviour that truly matters in the organisation’s context would need to be recovered. Response of the curricula to diverse contexts remains critical.

### Study limitations

The scoping review research is based on published articles. Some articles can be viewed as outdated, thus creating doubts about their relevance and applicability in the current period. The low number of articles that specifically address healthcare leadership development may also develop doubts about the representativeness of the research data.

## Conclusion

Healthcare LDPs exist in sub-Saharan Africa. However, their focus is more on healthcare management development, Context and needs-based healthcare leadership development, underpinned by healthcare-specific competency models, provides an essential cornerstone for designing strategically aligned healthcare LDPs. Effective leadership, which continuously improves health system performance, requires leadership models to be adapted for local contexts with a focus on healthcare leadership competency development.

### Implications and recommendations

By addressing the established gaps in healthcare leadership practices and implementing context and evidence-based interventions, policymakers and practitioners in sub-Saharan Africa can strengthen health systems, improve service delivery and ultimately enhance the population’s health outcomes.

Policymakers are advised to consider standardisation of curricula and institutionalisation of programmes if the impact of healthcare leadership development is to deliver the much-desired health system’s outcomes.

This scoping review provides a foundation for future research around healthcare LDPs localisation, standardisation and institutionalisation, as well as the actions needed to build a resilient and effective healthcare leadership in the region.
